# Native bluegill influence the foraging and aggressive behavior of invasive mosquitofish

**DOI:** 10.7717/peerj.6203

**Published:** 2019-01-10

**Authors:** Jennifer H. Clemmer, Jessica E. Rettig

**Affiliations:** Department of Biology, Denison University, Granville, OH, United States of America

**Keywords:** Mosquitofish, Gambusia, Aggression, Invasive species, Foraging, Bluegill, Lepomis, Behavior, Native fishes

## Abstract

Two fish species that are common invaders of aquatic ecosystems world-wide are *Gambusia affinis* and *G. holbrooki*, commonly known as mosquitofish. In North America, introduced *G. affinis* are thought to have contributed to the population decline of several native fish species. Sunfish (family Centrarchidae) naturally occur across much of North American, thus mosquitofish and sunfish are likely to come into contact and interact more frequently as mosquitofish spread. However, the nature of this interaction is not well known. We used a lab experiment to explore whether and how the aggressive and foraging behaviors of *G. affinis* might be influenced by a representative and ubiquitous native centrarchid (*Lepomis macrochirus*; bluegill sunfish), a species with juveniles that inhabit littoral habitats also preferred by mosquitofish. The experiment partnered an individual male or female mosquitofish (focal fish) with a juvenile bluegill, or a same- or opposite-sex conspecific, filmed these one-to-one interactions, and quantified foraging and aggressive actions for the focal mosquitofish. We found that juvenile bluegill affect foraging in male mosquitofish, resulting in lower percent of handling attempts and handling time in which the male consumed a food item. The presence of juvenile bluegill also led to a reduction in the number of aggressive acts by mosquitofish compared to aggression levels when focal mosquitofish were with conspecifics. In nature, when mosquitofish encounter juvenile bluegill in littoral habitats, our results suggest that the foraging and aggressive behaviors of mosquitofish will be modified, especially for males. This mechanism may influence the rate or geographic extent of the spread of mosquitofish into North American waterbodies.

## Introduction

Mosquitofish (*Gambusia affinis and G. holbrooki*) are native to eastern North America and the Mississippi River drainage, but have been successfully introduced to or have invaded aquatic ecosystems on every continent except Antarctica (see reviews by Pyke, 2005; Pyke, 2008). Whether by introduction or invasion, multiple studies show that the presence of mosquitofish negatively impacts native fishes, causing reductions in population size or local population elimination (see reviews by Arthington & Lloyd, 1989; Courtenay & Meffe, 1989; Pyke, 2008).

Within its native range *G. affinis* coexists with many fish species, and river and stream surveys indicate that the relative abundance of *G. affinis* does not affect the species richness or diversity of the native fish assemblage (Matthews & Marsh-Matthews, 2011). In contrast, the entry of *G. affinis* into the habitats of other North American native fishes has been followed by declines in population size for the Barrens topminnow in Tennessee (*Fundulus julisia*; Laha & Mattingly, 2007), the least chub in Utah (*Iotichthys phlegethontis*; Mills, Rader & Belk, 2004), and the plains topminnow (*Fundulus sciadicus*; Schumann, Hoback & Koupal, 2015), and the local elimination of Arizona populations of the Sonoran topminnow (*Poeciliopsis occidentalis*; Meffe, 1983; Meffe, 1985). Thus, for some North American native fishes, the potential exists for mosquitofish to negatively affect their abundance.

The success of mosquitofish outside their native range has been attributed to multiple factors, including their foraging and aggressive behaviors, such that in experiments testing foraging ability, both *G. affinis* and *G. holbrooki* ate more prey and foraged with greater efficiency than did *Gambusia* species which are not invasive (Rehage, Barnett & Sih, 2005a; Rehage, Barnett & Sih, 2005b). In addition, mosquitofish often are characterized as very aggressive toward other fishes; *G. affinis* routinely nipped the caudal fins of juvenile inanga (*Galaxias maculatus*) ultimately resulting in death (Rowe, Smith & Baker, 2007). Aggressive behaviors or direct predation by *G. affinis* on native species has also been shown for black mudfish (*Neochanna diversus*) (Barrier & Hicks, 1994), Barrens topminnow (Laha & Mattingly, 2007), least chub (Mills, Rader & Belk, 2004), northern starhead topminnow (*Fundulus dispar*) and banded killifish (*Fundulus diaphanous*; (Sutton, Zeiber & Fisher, 2013), and plains topminnow and northern plains killifish (*Fundulus kansae*) (Schumann, Hoback & Koupal, 2015). These studies suggest that the success of mosquitofish invasions may be related to the vigor of their foraging and aggressive behaviors toward fishes naturally present in the system.

Given that mosquitofish are often successful when newly entering a water body and that the vigor of mosquitofish behavior may contribute to this success (i.e., population establishment and growth), we wanted to explore whether and how a native fish might influence these behaviors, thus possibly ameliorating the negative effects mosquitofish have on some species in native fish assemblages. We selected the bluegill sunfish (*Lepomis macrochirus*) as our representative native fish because they are native to eastern North America and have been introduced widely west of the Rocky Mountains (Berra, 2001). The broad distribution of bluegill (and other lepomids) make it increasingly likely that mosquitofish will invade *Lepomis* habitats. If the presence of lepomids prompts mosquitofish to forage differently or behave less aggressively, then sunfish may help constrain mosquitofish spread or population growth.

In many ponds and lakes bluegill typically occur in dense populations (Ehlinger, 1989) wherein juveniles spend 2–4 years in the littoral zone, foraging on zooplankton and macroinvertebrates before sexual maturity (Mittelbach, 1981; Werner & Hall, 1988), but see alternative life history pattern reported by (Gross, 1982; Neff, Cargnelli & Côté, 2004). Adult bluegill are generalist predators, feeding on a variety of prey with an emphasis on zooplankton (Ehlinger, 1989; Mittelbach, 1981; Werner & Hall, 1988). Bluegill are also adaptable predators in that they can alter their foraging to contend with seasonal changes in the prey assemblage (Werner et al., 1983). By frequenting the littoral zone, juvenile bluegill occupy the same habitat used by mosquitofish (reviewed in Pyke, 2005).

Western mosquitofish (*G. affinis*) occur in high densities in shallow, vegetated littoral zones (Pyke, 2008), where they actively feed on zooplankton, aquatic invertebrates, and the adults of some aquatic insects (Crivelli & Boy, 1987; García-Berthou, 1999; Pyke, 2005). As such, the diet overlap between juvenile bluegill and mosquitofish is likely to be substantial, resulting in inter-specific competition. Foraging by *G. affinis* is also influenced by intra-specific agonistic interactions between males and females, such that both males and females in mixed-sex groups exhibited fewer prey strikes compared those in single-sex groups (Arrington et al., 2009), and females in groups foraged longer when males were absent (Plath et al., 2007). Thus in assessing how juvenile bluegill affect mosquitofish foraging, one must also be aware that social interactions between female and males may alter behaviors.

Our study used a lab experiment, in which we paired a single mosquitofish with a juvenile bluegill, a male mosquitofish, or female mosquitofish, to examine whether the identity of the partner fish influenced the behavior of the focal mosquitofish. We hypothesized that the presence of juvenile bluegill might alter the behavior of adult mosquitofish. Because the foraging behavior of mosquitofish is also complicated by male–female interactions, our experiment used gender of the focal fish as a main effect, along with the main effect of partner identity described earlier. Specifically we asked, does the behavior of a focal male or female mosquitofish change in the presence of bluegill compared to their behavior in the presence of a conspecific? If mosquitofish do alter their behavior in the presence of juvenile bluegill, then this could affect the ability of mosquitofish to invade or/and maintain large populations therein.

## Methods

### Fish collection and maintenance

We dip-netted male and female adult mosquitofish (*G. affinis*) from the vegetated littoral zone of Olde Minnow Pond located on the Denison University Biological Reserve (40°5′N, 82°31′W) in Licking County, Ohio on June 1 (i.e., early in the breeding season). *G. affinis* were introduced to this pond sometime prior to 1981 (USGS, 2018). Female mosquitofish in this pond were significantly larger than males (ANOVA: *F*_1,58_ = 99.97, *P* < 0.0001; females 33.8 ± 0.5 mm, males 27.4 ± 0.4 mm (mean ± SE total length)). We collected small juvenile bluegill (i.e., non-reproductive; mean ± SE total length: bluegill 41.6 ± 0.8 mm) by seining the littoral zone of nearby Middleton Pond in Licking County, Ohio (40°3.36′N, 82°32.55′W) on June 3, a pond containing bluegill as the only lepomid. Although body size differed among juvenile bluegill, female mosquitofish, and male mosquitofish, we collected and used these naturally co-occurring sizes to mimic the encounters that exist in the littoral zone.

In the laboratory, we acclimated the fish to a standard 21 °C temperature and used separate aquaria to house male mosquitofish, female mosquitofish, and bluegill, holding no more than 20 fish per aquaria. Each aquaria (51 cm long × 26 cm wide × 32 cm tall) was filled with 37 liters of conditioned tap water, and provided an external filtration system (Whisper 20 Power Filter^®^), and two airstones. No shelters were provided for the fish because none would be available in the experimental trials. Twice a day we added freeze-dried bloodworms (*Chironomus* spp.; Tetra^®^) and commercial food flakes (TetraMin; Tetra^®^) to the aquaria so fish could feed; we removed excess food once fish stopped eating. Water in these maintenance tanks was refreshed twice a week by removing 1/3 of the water and replacing it with conditioned tap water. Prior to each behavioral trial, all fish were deprived food for 24 h, then we removed fish to be used in the upcoming trial and resumed routine feeding in all maintenance aquaria.

### Behavioral trials

Twenty-four hours before each experimental trial, we removed fish from the maintenance aquaria and placed them in white opaque plastic bins (28 cm long ×15 cm wide ×11 cm tall) filled with 3 L aged, oxygenated tap water, where the fish acclimated without food overnight. Experimental fish thus were deprived of food for 48 h to increase the likelihood that fish would feed during the trial. The bins did not have an airstone, because water movement caused by the airstones prevented seeing the fish on film, but fish exhibited no signs of oxygen limitation (e.g., gasping or ventilating at the surface (aquatic surface respiration), odd swimming patterns, or loss of appetite). A clear plastic divider across the center of each bin physically separated the two fish, but perforations in the divider potentially allowed for chemical and visual cues to be exchanged. To reduce disturbance from activities in the lab, the array of five experimental bins was screened on the sides and top using cardboard. Filming (real time) occurred through a hole in the cardboard above each bin, using a Sony Handycam^®^ DCR-SX63 mounted on a tripod.

Our experiment manipulated two main effects: partner identity (i.e., bluegill, same-sex mosquitofish, and opposite-sex mosquitofish) and gender of the focal mosquitofish (male or female) to analyze the response of a focal mosquitofish to these main effects and their interaction. Each experimental trial involved the sequential filming of five bins, each of which contained one of the five pairings in our study that crossed partner identity with gender of the focal fish in the following combinations:

1 ♂ mosquitofish + 1 bluegill

1 ♀ mosquitofish + 1 bluegill

1 ♂ mosquitofish + 1 ♂ mosquitofish

1 ♀ mosquitofish + 1 ♀ mosquitofish

1 ♂ mosquitofish + 1 ♀ mosquitofish.

We conducted 10 trials, yielding 10 replicates of each pairing, except for the pairing of a male and female mosquitofish. This pairing had five replicates for a focal male and five replicates for the focal female to avoid pseudoreplication in the bins containing one male and one female mosquitofish due to a lack of independence in the response of each focal fish to the opposite-sex fish. We filmed each trial between 09:00 and 12:00 and only filmed one trial per day; each trial was separated by 3–4 days. Fish were only used once during the entire experiment. In the first trial the pairings were randomly assigned to one of the bins and the bins were sequentially filmed in numerical order. In subsequent trials, the pairings rotated to the next bin numerically, but the bins continued to be filmed sequentially in numerical order. This resulted in each pairing being filmed at each possible time and in each bin position, thus reducing the chances that time or position of the bin in the array influenced the results.

During an experimental trial we filmed each bin for 15 min. Filming began when the plastic divider was carefully lifted up from the bin to minimize disturbance to other bins in the array and 0.04 g of freeze-dried bloodworms (*Chironomus* spp.; Tetra^®^) were added to the center of the bin, a food item that initially floats and one to which the fish consumed in the maintenance aquaria. Once all bins were filmed in a trial we measured both fish (total length, mm) from each bin, rather than measuring the fish before the trials and possibly causing pre-experiment stress.

We viewed the recording of each pairing in each trial to identify and count the number and duration of foraging behaviors for individual fish. We also quantified the aggressive acts occurring between the fish in each treatment. We used a focal animal approach to collecting the data, focusing on the behaviors of a single fish at a time. For treatments with two male or two female mosquitofish the behavior of only one focal individual was quantified, so that comparisons among all pairing were based on the behavior of a single fish. We quantified behavior for 10 min of the recording period to permit a few minutes for the fish to adjust following the removal of the plastic separation barrier; we commenced data collection with the focal fish’s first strike at a food item. This focused our observations to a standard time period (10 min) when each focal fish was highly engaged in feeding and any associated aggressive acts.

A foraging attempt was defined as beginning when the fish oriented its body toward the food item in preparation for striking and ending when the fish either swallowed or released the food. The small size of the fish prevented us from detecting when the eyes initially oriented toward the food. Each foraging attempt was classified as successful if the fish swallowed the food item and unsuccessful if the fish released the food item. When the fish’s position relative to the camera allowed us to clearly visualize the fish handling its food (i.e., food manipulation in the mouth; jaws moving), we also quantified the number of handling attempts and the time spent manipulating the food item in the mouth (handling time). Handling attempts and handling time were classified as successful if the food was swallowed or unsuccessful if the food item was released back into the water.

Aggressive behaviors included intention movements, chases, and nips, defined as per Laha & Mattingly (2007) and Barrier & Hicks (1994):

• Intention movement: a brief (<1s) action where one fish quickly turns towards another fish or makes a very short advance towards another fish propelled by its tail or pectoral fins;
• Chase: where one fish quickly swims after the other fish for >1s with the fish being in close proximity (≤10 cm head to head);
• Nip: physical contact directed from one fish to the other such as pushing or biting.

These behaviors were quantified when the focal fish produced the behavior or when the behavior was targeted toward the focal fish.

### Data analysis

We employed two-way ANOVA (JMP^®^ Pro 12; SAS Institute, Inc., Cary, N.C.) to compare the behavior of focal mosquitofish in response to the main effects of partner identity (i.e., juvenile bluegill, opposite-sex conspecific, or same-sex conspecific) and gender (i.e., focal fish is male or female) and the interaction of these effects. We analyzed foraging variables including number of foraging attempts, total foraging time, number of handling attempts, and total time spent handling a food item. Additionally, we were interested in knowing the percentage of attempts or time spent by the focal fish that resulted in successful food consumption as a way to see if the focal fish had differential foraging success with partner identity or gender. Thus, we calculated for each focal fish the proportion of total attempts or proportion of total time that resulted in successful consumption of a food item, and then analyzed these data. Finally we analyzed the total number of aggressive acts performed by the focal fish and the total number of aggressive actions directed at the focal fish. In our analyses, proportional data (e.g., proportion of foraging attempts that were successful) were arcsin square-root transformed prior to analysis and data were log(x+1) transformed when needed to meet the assumptions of homogeneity of the variances (Ott, 1988).

## Results

### Foraging behaviors

#### Foraging attempts

Although focal mosquitofish could be partnered with a bluegill, or a same or opposite sex mosquitofish, the number of foraging attempts made by focal fish (Table 1) was not affected by partner identity (*F*_2,43_ = 2.00, *P* = 0.15), gender of the focal fish (*F*_1,43_ = 0.53, *P* = 0.47), or their interaction (*F*_2,43_ = 0.18, *P* = 0.83). Focal mosquitofish also showed no difference in the proportion of foraging attempts that were successful according to partner identity (*F*_2,43_ = 0.11, *P* = 0.90), gender (*F*_1,43_ = 0.59, *P* = 0.45), or the interaction of these main effects (*F*_2,43_ = 1.68, *P* = 0.20).

#### Foraging time

**Figure 1:**
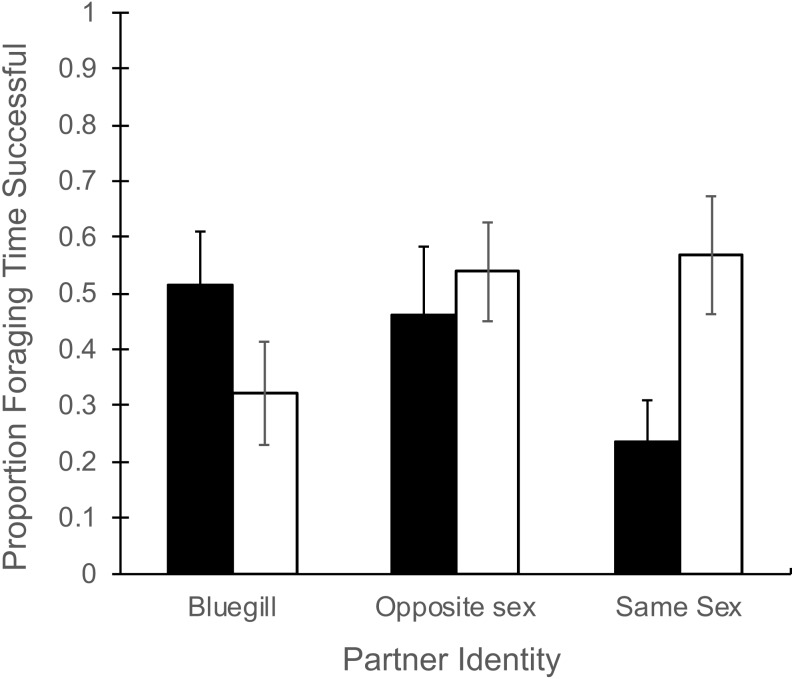
Proportion of foraging time resulting in food consumption for male and female mosquitofish. Mean proportion of foraging time (±1 SE) that focal fish successfully consumed a food item for male (black bars) and female (white bars) mosquitofish according to the identity of the focal fish’s partner: bluegill, opposite sex mosquitofish, or same sex mosquitofish. *N* = 10 for bluegill and same sex, and *N* = 5 for the opposite sex.

Total time spent foraging (Table 1) did not depend on partner identity (*F*_2,43_ = 0.51, *P* = 0.60), and there was no effect of gender (*F*_1,43_ = 0.61, *P* = 0.44) or the interaction of partner and gender (*F*_2,43_ = 1.29, *P* = 0.28). There was a significant interaction of partner identity with gender for the proportion of foraging time that resulted in successful food consumption (Fig. 1, *F*_2,43_ = 4.32, *P* = 0.02). In particular, when a focal male mosquitofish was partnered with another male only 24% of foraging time led to success, in contrast to 56% success when a focal female partnered with another female (Fig. 1). The main effects of partner identity (*F*_2,43_ = 0.26, *P* = 0.77) or gender (*F*_1,43_ = 0.83, *P* = 0.37) had no effect on the proportion of foraging time that was successful.

**Table 1:** Foraging behaviors of male and female mosquitofish according to gender of the focal mosquitofish and partner identity. Mean (±1 SE) for the number of foraging attempts, total foraging time, number of handling attempts, and total handling time, split by gender of the focal mosquitofish and partner identity. Note that total handling time sometimes exceeds total foraging time because we had fewer observations of handling time, but for any individual fish the total foraging time always was greater than total handling time.

			Partner identity		
			Bluegill	Opposite sex	Same sex
Gender	♀	# Foraging attempts	12.3 ± 3.0	6.8 ± 1.0	9.0 ± 1.4
	♂	# Foraging attempts	9.8 ± 2.0	7.0 ± 2.5	7.5 ± 1.3

Gender	♀	Total foraging time (s)	126.0 ± 44.8	29.10 ± 7.90	56.59 ± 19.5
	♂	Total foraging time (s)	81.30 ± 42.1	84.97 ± 60.26	129.72 ± 47.5

Gender	♀	# Handling attempts	2.57 ± 0.6	1.50 ± 0.50	2.25 ± 0.9
	♂	# Handling attempts	1.3 ± 0.3	2.0 ± 1.0	2.0 ± 0.7

Gender	♀	Total handling time (s)	104.5 ± 49.5	17.92 ± 2.68	78.6 ± 33.3
	♂	Total handling time (s)	143.7 ± 134.5	141.2 ± 132.9	144.4 ± 56.6

#### Handling attempts

**Figure 2:**
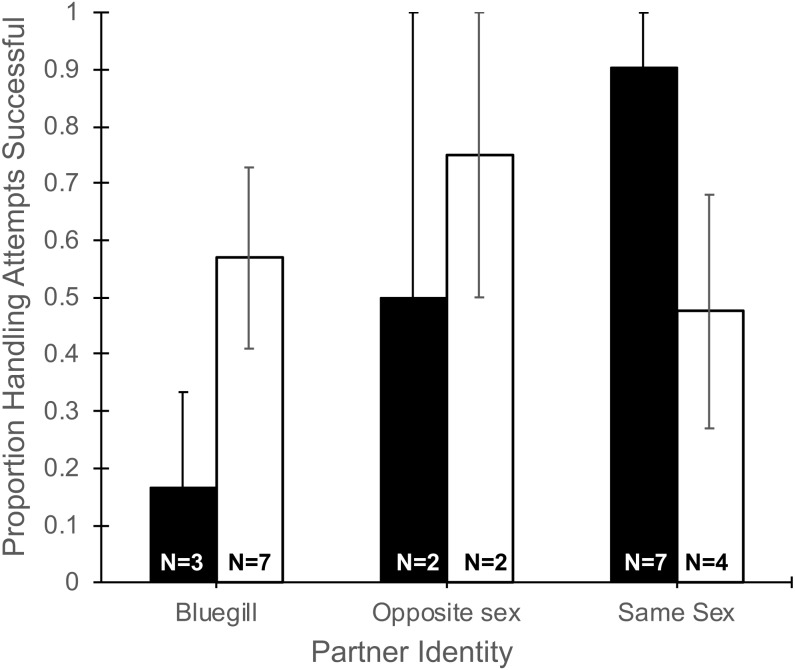
Proportion of handling attempts that were successful for male and female mosquitofish Mean proportion of the total handling attempts (±1 SE) that were successful for male (black bars) and female (white bars) mosquitofish according to the identity of the focal fish’s partner: bluegill, opposite sex mosquitofish, or same sex mosquitofish. A handling attempt was successful if the focal fish consumed the food item. Sample size is indicated on the bars for each pairing.

The number of handling attempts by focal mosquitofish (Table 1) did not differ based on partner identity (*F*_2,19_ = 0.09, *P* = 0.92), gender (*F*_1,19_ = 0.21, *P* = 0.65), or their interaction (*F*_2,19_ = 0.05, *P* = 0.64). Partner identity interacted with gender to produce a marginally significant trend (*F*_2,19_ = 3.35, *P* = 0.057, Fig. 2) in the proportion of handling attempts that ended in successful consumption of food. Notably, focal males partnered with a bluegill were successful in 16% of their handling attempts, much lower than other pairing combinations, and in sharp contrast to the 90% success in handling attempts for a focal male paired with another male (Fig. 2). Differences in partner identity (*F*_2,19_ = 1.76, *P* = 0.20) or gender (*F*_1,19_ = 0.24, *P* = 0.62) alone did not affect the proportion of handling attempts that were successful.

#### Handling time

**Figure 3:**
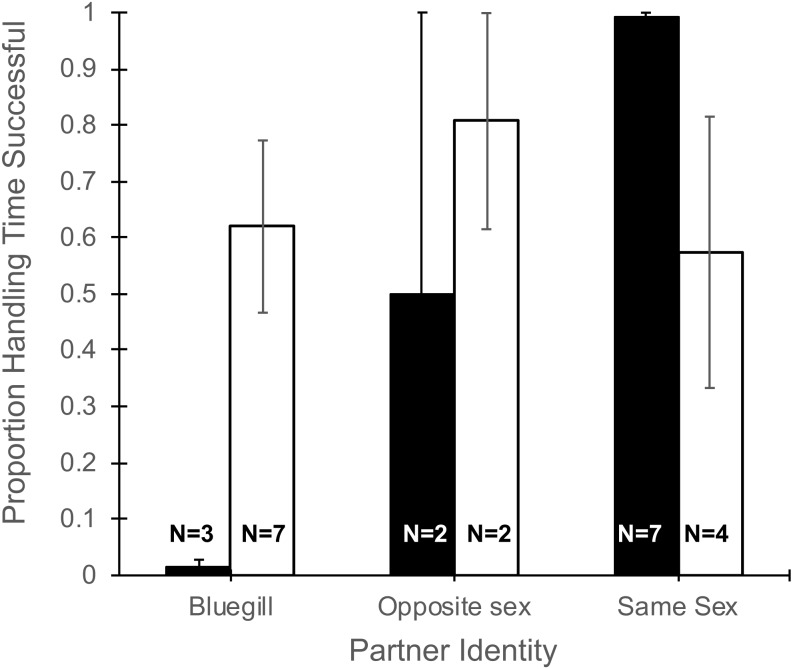
Proportion of total handling time resulting in successful consumption of food for male and female mosquitofish. Mean proportion of handling time (±1 SE) that was successful for male (black bars) and female (white bars) mosquitofish according to the identity of the focal fish’s partner: bluegill, opposite sex mosquitofish, or same sex mosquitofish. Handling time was successful if the focal fish consumed the food item. Sample size is indicated on the bars for each pairing.

The total time a focal fish spent handling a food item (Table 1) did not differ with partner identity (*F*_2,19_ = 0.13, *P* = 0.88), gender of the focal fish (*F*_1,19_ = 1.34, *P* = 0.26), or their interaction (*F*_2,19_ = 0.12, *P* = 0.89). However, the proportion of time spent handling food that was consumed was effected by the interaction of partner identify and gender (*F*_2,19_ = 5.27, *P* = 0.015, Fig. 3). Specifically, focal males paired with bluegill had the lowest percentage of handling time that was successful (1.4%) and males paired with another male had the highest success (99%), while focal females showed little difference in the proportion of handling time that was successful whether partnered with bluegill or conspecifics (Fig. 3). Additionally, the main effect of partner identity also was significant (*F*_2,19_ = 3.90, *P* = 0.038) such that when bluegill were present the proportion of handling time resulting in success was roughly half (0.44 ± 0.14, mean ± 1 SEM) that of pairings with a same-sex conspecific (0.84 ± 0.10), but there was no effect of gender (*F*_1,19_ = 0.98, *P* = 0.34).

### Aggressive behaviors

**Figure 4:**
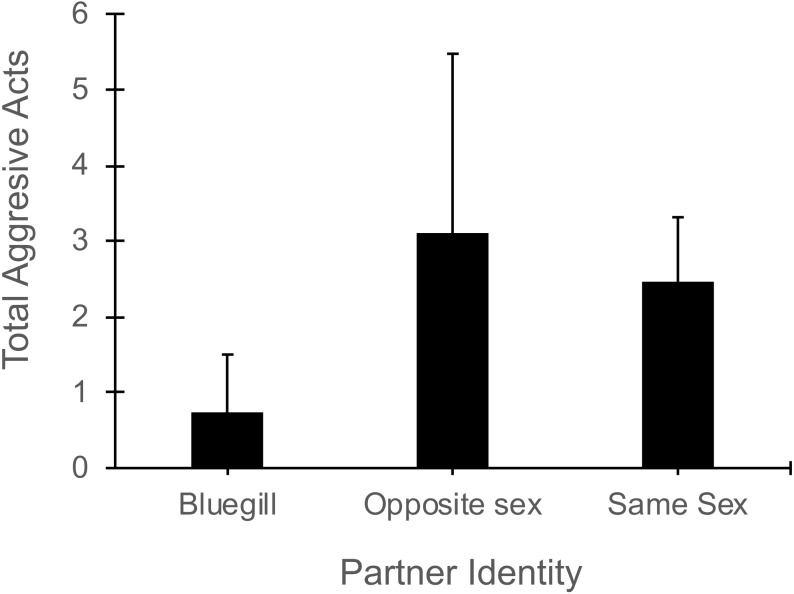
Number of aggressive acts made by focal mosquitofish. Mean number of aggressive acts (±1 SE) made by focal mosquitofish according to the identity of the partner: bluegill, opposite sex mosquitofish, or same sex mosquitofish. *N* = 10 for bluegill and same sex, and *N* = 5 for the opposite sex.

In the presence of a bluegill, focal mosquitofish reduced the total number of aggressive acts (i.e., sum of nips, chases, and intention movements) they exhibited compared to their aggression in the presence of same-sex or opposite sex conspecifics (Partner Identity effect: *F*_2,44_ = 3.40 , *P* = 0.04, Fig. 4). The number of aggressive acts by the focal fish was not affected by gender (*F*_1,44_ = 1.70, *P* = 0.20) or the interaction of partner identity with gender (*F*_2,44_ = 2.17, *P* = 0.13). The number of aggressive acts directed toward a focal mosquitofish was similar across partner identity (*F*_2,44_ = 9.4 , *P* = 0.39) and between genders (*F*_1,44_ = 2.54, *P* = 0.12), and the interaction of partner identity and gender was not significant (*F*_2,44_ = 0.62, *P* = 0.60).

## Discussion

Mosquitofish are a successful and prolific invasive species, consequently they are likely to interact with native fishes in the locales they colonize. Our study asked whether and how the presence of native juvenile bluegill influenced the foraging or aggressive behaviors of the non-native mosquitofish.

### Bluegill influence mosquitofish foraging

Because the diets of mosquitofish and bluegill overlap, how the species interact behaviorally may affect population success when they co-occur (either naturally or following invasion). We found that the percent of successful handling attempts and percent time spent handling food successfully was lower for male mosquitofish, but not females, when juvenile bluegill were present. Thus, bluegill had a negative effect on some aspects of foraging in male mosquitofish, while the foraging of female mosquitofish was more resistant to the influence of native bluegill. To date, how or why the feeding behavior of mosquitofish is affected by other fish species has received little attention. Using lab experiments Rehage, Barnett & Sih (2005a) found, similar to our findings, that *Gambusia affinis* altered their foraging behavior in the presence of another fish species (fathead minnow, *Pimephals promelas*), specifically mosquitofish consumed fewer prey but foraged at a higher rate (# prey/minute) when *P. promelas* was present compared to when it was absent.

Our findings suggest that when mosquitofish in nature enter waterbodies containing juvenile bluegill, they are apt (males more so than females) to obtain fewer resources (i.e., successfully handle and swallow fewer prey) foraging in the vicinity of a bluegill compared to foraging with conspecifics. Reduced feeding success is likely to lower mosquitofish fitness, affect individual and population growth and size, impact recruitment, and influence competition with native fishes. In other words, the presence of bluegill in habitats that mosquitofish invade may alter if, how, and when mosquitofish continue to spread.

### Conspecific influence on mosquitofish foraging

In addition to interacting with native fish, mosquitofish also encounter conspecifics in nature making it important to consider how behavioral interactions between conspecifics might affect foraging success. In particular, mosquitofish may forage differently in the presence of same-sex versus opposite-sex conspecifics, due to agonistic interactions between males and females (e.g., Arrington et al., 2009; Plath et al., 2007). Our study found that male and female fish with a same-sex partner differed in the percent of foraging time that was successful (females > males) and differed in the percent of handling attempts and handling time that was successful (males > females), however there was no difference in these variables when each gender was partnered with the opposite sex. Thus gender interacts with the identity of the partner fish to affect foraging behaviors in mosquitofish, and it appears that same-sex interactions produce different results for males and females, while opposite-sex interactions do not.

For conspecific interactions, our findings contrast with those of Heubel & Plath (2008) and Pilastro, Benetton & Bisazza (2003), who found in mosquitofish or related poeciliids that female foraging (e.g., feeding times and/or other foraging parameters) is compromised by the presence of males due to male reproductive harassment. One explanation for the difference in our outcomes is that in our study both fish were denied food prior to the experimental trial, whereas in other studies only the focal fish was hungry while the partner was satiated. This procedural difference suggests that the female mosquitofish in our study may not have been bothered when paired with conspecific males because the males were more intent on foraging during the trial. Nonetheless, the fact that our study showed that female foraging does not differ significantly when in the presence of either males or bluegill suggests that females may be better able to tolerate the consequences of sharing a habitat with competitors.

### Body size effects

The response of male mosquitofish to bluegill and the lack of response by female mosquitofish may reflect body size rather than species identity. The male mosquitofish and juvenile bluegill we collected in the ponds differed in size (27.4 mm vs. 41.6 mm total length respectively), and smaller fishes are known to alter their behaviors in the presence of larger fishes, in some cases reducing their foraging activity or changing their foraging location (Hjelm & Persson, 2001; Rincón et al., 2002; Schulze et al., 2006). That said, our male mosquitofish were smaller than female mosquitofish (33.8 mm total length), but male foraging behavior was unaffected by the larger females (indeed it appears that hunger levels might influence behaviors, see above). In addition, female mosquitofish (while larger than males) were smaller than the juvenile bluegill, but their behavior remained unaltered by partner identity (larger or smaller partners). Thus in our study differences in body size alone are unlikely to fully explain the behavioral responses of focal mosquitofish to other fish, as gender and species identity also are a likely influence. While it is appealing to contemplate why male mosquitofish alter their foraging behavior in the presence of larger juvenile bluegill or how the results might differ if partners were equal sized, the important finding is that male mosquitofish do alter their behavior and do so in ways that in nature might hamper fitness, population growth, or recruitment.

## Aggression

As seen with foraging, few studies have explored how mosquitofish aggression is effected by the presence of a heterospecific. Some show that mosquitofish aggression is greater than aggression by native species, including the Iberian toothcarp (*Aphanius iberus*), plains topminnow (*F. sciadicus*), northern plains killifish (*F. kansae*), and northern starhead topminnow (*F. dispar*) (see Carmona-Catot, Magellan & García-Berthou, 2013; Schumann, Hoback & Koupal, 2015; Sutton, Zeiber & Fisher, 2013) or exceeds aggression of native fish which are naive to mosquitofish (ornate rainbowfish, *Rhadinocentrus ornatus*; (Keller & Brown, 2008). Thus, our finding of mosquitofish reducing their aggressive behavior in the presence of native juvenile bluegill is an important contribution.

As with the case of foraging, the changes in aggressive behaviors in mosquitofish could be due in part to body size differences. Size-dependent interactions are common among fishes: in the presence of larger fish, smaller fishes may experience predation (Schröder et al., 2009), competition for prey (Deaton, 2008; Natsumeda, Mori & Yuma, 2012; Grabowska et al., 2016), or experience higher levels of aggression from large fish (Imsland et al., 2016; Yue, Duncan & Moccia, 2006; Magellan & García-Berthou, 2015). Given that mosquitofish are notably aggressive (Pyke, 2008), and that their aggression is thought to contribute to their invasiveness, our finding that juvenile bluegill moderate mosquitofish aggressiveness suggests that bluegill may diminish the degree to which mosquitofish impact or invade native fish communities.

Because our experiment tested only juvenile bluegill, we cannot say how mosquitofish might also alter their foraging or aggressive behavior in the presence of yet larger bluegill (i.e., adults). However, we anticipate that changes would indeed be seen, given that larger fish (hetero- or conspecifics), routinely affect the behavior of smaller fishes through aggression, competition, or changes in habitat use (Davey et al., 2005; Schulze et al., 2006), any of which might function to minimize contact, reduce interaction, minimize competition, and potentially lead to continued co-existence. In addition, not to be ignored is the effect of direct predation of small fish by larger ones (hertero- or conspecifics). For example, laboratory and mesocosm experiments have shown that adult sunfish (*L. macrochirus, L. cyanellus, L. gulosus, L. megalotis*) prey upon and thus influence if and how invasion or re-invasion by smaller minnow species occurs (Marsh-Matthews, Matthews & Franssen, 2011; Marsh-Matthews et al., 2013); however, mosquitofish were not tested by these authors. Although questions of if and how larger adult bluegill affect the behavior of mosquitofish and the dynamics of the mosquitofish populations remain to be examined, our study demonstrates that native juvenile bluegill influence both foraging and aggressive behaviors of non-native mosquitofish.

##  Supplemental Information

10.7717/peerj.6203/supp-1 Supplemental Information 1 Focal fish foraging and aggressive behaviors in response to five experimental treatments

## Additional Information and Declarations

### Data Availability

The following information was supplied regarding data availability: The raw data available in the Supplemental File shows the foraging and aggressive response variables for the focal fish in each replicate of each treatment.
